# Immune cell pathology in rabbit hemorrhagic disease

**DOI:** 10.14202/vetworld.2019.1332-1340

**Published:** 2019-08-28

**Authors:** Anna Babken Semerjyan, Mariam Armenak Sargsyan, Hranush Harutyun Arzumanyan, Lina Hayrapet Hakobyan, Liana Onik Abroyan, Zara Babken Semerjyan, Aida Sergey Avetisyan, Elena Michael Karalova, Davit Mihran Manukyan, Hripsime Shavarsh Matevosyan, Nikolay Fyodor Krasnikov, Zaven Alexandr Karalyan

**Affiliations:** 1 Department of Medical Biology, Yerevan State Medical University, Yerevan, Armenia; 2 Department of Epidemiology and Parasitology, Armenian National Agrarian University, Yerevan, Armenia; 3 Laboratory of Cell Biology and Virology, Institute of Molecular Biology of NAS RA, Yerevan, Armenia

**Keywords:** cytopathology, emperipolesis, eosinophilic viral inclusions, immune response, macrophages, rabbit hemorrhagic disease virus

## Abstract

**Aim:**

The aim of this research was to study the effect of rabbit hemorrhagic disease virus (RHDV) on the host immune response by examining the cellular composition/pathology of lymphoid organs and serum levels of tumor necrosis factor-alpha (TNF-α) and interferon-gamma (IFN-γ).

**Materials and Methods:**

Nine adult rabbits were inoculated with 1 ml of 10% infected liver homogenate, and three rabbits served as controls. The rabbit hemorrhagic disease (RHD)-induced animals were studied on 3 consecutive days post-infection. Diagnosis of RHD was made through routine hemagglutination tests and the polymerase chain reaction. Blood smears and tissue samples from bone marrow (BM), spleen, lymph nodes, and liver were analyzed for cell composition and cytopathology. Serum levels of TNF-α and IFN-γ were measured by enzyme-linked immunosorbent assay.

**Results:**

RHD showed a decreased absolute cell count of blood as well as lymph nodes, spleen, and BM cell populations with marked left shift. This was seen as a progressive rise in immature and blast cells. Quantitative cellular changes were accompanied by an increase in specific inflammatory cytokines. Immunocytopathological alterations were evidenced by: Vacuolized, hyperactivated tissue macrophages, finding of Döhle bodies in neutrophils, and activated lymphocytes with increased nuclear-cytoplasmic ratio. Cytoplasmic eosinophilic viral inclusions found in tissue (liver, spleen, and BM) macrophages were shown for the 1^st^ time in RHD. Megakaryocytic emperipolesis was a common feature of RHD.

**Conclusion:**

These studies suggest that RHDV induces pathology in leukocytes due to hyperactivation with left shift (toward immature stages of the different cell lineages). Macrophages are increased in number and show an expressed cytopathic effect often accompanied by viral eosinophilic cytoplasmic inclusions. They also developed a secretory activation (increased levels of pro-inflammatory cytokines).

## Introduction

As small mammals, rabbits are still found both in the wild and as domestic pets in several countries such as Spain, Italy, and Egypt [1]. They have a high reproductive rate which helps solve, in part, the world food shortage problem. Understanding more about factors that can influence animal welfare is extremely important to improve the well-being of high-quality products [2]. Rabbit hemorrhagic disease (RHD) is one of these factors. RHD is often a lethal and extremely contagious viral disease of wild (*Oryctolagus cuniculus*) and domestic (*O. cuniculus domesticus*) rabbits [3]. It is caused by an RNA virus of the family *Caliciviridae*, genus *Lagovirus* – *Lagovirus europaeus* GI.1 (previously called rabbit hemorrhagic disease virus [RHDV]) and *L. europaeus* GI.2 (previously called RHDV2 or RHDVb) [4-6]. RHD is characterized by acute liver damage and disseminated intravascular coagulation [7,8]. Although the first outbreak of RHD was more than 30 years ago, the mechanisms underlying its pathogenesis are still not fully understood. The liver is believed to be the main site of RHDV reproduction, with viral replication leading to liver cell apoptosis and necrosis [7]. Systemic hemorrhagic diathesis and disseminated intravascular coagulation can lead to mortality. These are most likely the consequences of liver cell loss through RHDV-induced apoptosis [8]. A substantial contributing factor in the development of viral hemorrhagic fever is also the pathology of the immune system. Studies have shown that macrophages and endothelial cells display morphological hallmarks of apoptosis [9].

Further research has found that also granulocyte and lymphocyte apoptosis occurs in rabbits infected with various RHDV strains [10]. Cytokines play an important role in the regulation of the immune response and the pathogenesis of many diseases, including those caused by viral infections. Studies by Trzeciak-Ryczek *et al*. [11,12] indicate a role for the immune response, especially the peripheral blood leukocytes, in the pathogenesis of RHD. Studies of immunopathology in rabbit hemorrhagic viral disease could provide a useful model for the study of various human pathologies [13].

The aim of this research was to study the effect of RHDV on the host immune response by examining the cellular composition/pathology of lymphoid organs and serum levels of tumor necrosis factor-alpha (TNF-α) and interferon-gamma (IFN-γ).

## Materials and Methods

### Ethical approval

Animal care and euthanasia were approved according to the American Veterinary Medical Association Guidelines on Euthanasia, and local guideline for animal care and use (Institutional Review Board/Independent Ethics Committee of the Institute of Molecular Biology of National Academy of Sciences, Yerevan, Armenia; IRB00004079, 2013).

### Virus

RHDV was obtained from the Agricultural University of Armenia. The virus was prepared from infected rabbit livers (10% homogenate). The hemagglutination (HA) test [14] and polymerase chain reaction (PCR) [15] were used for the diagnosis of RHD.

### Experimental animals

Twelve male rabbits (bucks), Armenian commercial breed, 6 months old, with an average weight of 2 kg, were purchased from a rabbitry. Before starting the experiments, the rabbits were kept for a 30 days quarantine period. All rabbits were RHDV-negative, confirmed by the HA inhibition test and PCR. Nine rabbits were inoculated (intramuscular injection) with 1 ml of 10% infected liver homogenate [16], and three rabbits served as controls.

Experimental animals were housed in separate cages and received standard care according to the Principles of Laboratory Animal Care. The vivarium temperature was 22-25°C. During the experiments, rabbits were carefully monitored to reduce stress if any.

### Blood smears, Giemsa staining, and white blood cells analysis

All blood samples were collected from the marginal ear vein. Pre-infection blood smears were used to control hematological values. Fresh blood was used in preparing the blood smears by routine methods. For nucleated blood cells analysis, slides were fixed in pure methanol and stained by Giemsa modified solution (azure B/azure II, eosin and methylene blue) according to the manufacturer’s protocol (Sigma-Aldrich), briefly: A solution of azure B/azure II-eosin/methylene blue 1:12:2 (w/w/w) in glycerol/methanol 5:24 (v/v), total dye content: 0.6% (w/w). Before use, the Giemsa solution was filtered and diluted (1:20) with buffer solution pH 6.5. Slides were fixed for 10 min in methanol, immersed for 45 min in Giemsa solution and rinsed with distilled water. White blood cells were examined under the light microscope at ×1250 in a random sequence. At least 200 white blood cells in each sample were evaluated for cell types.

Cell sizes were determined by routine cytometry using ImageJ software, open platform for scientific image analysis ((image processing program developed at the National Institutes of Health and the Laboratory for Optical and Computational Instrumentation (LOCI, University of Wisconsin, USA)).

### Tissue samples

At least five samples from bone marrow (BM), lymph nodes, spleen, and liver were taken from each animal and fixed in 10% buffered formalin solution (pH 7.2) for 24 h. After fixation, the samples were dehydrated through a graded series of alcohols (70%, 80%, 96%, and 100%), washed with xylol and embedded in paraffin wax. For morphological analysis, wax-embedded samples were cut 5 µ (Microm HM 355, Thermo Scientific) and stained with routine methods for hematoxylin and eosin (HE). Histological examinations were made using a light microscope (BOECO, BM-800, equipped with camera B-CAM10, Germany).

### Blood collection and enzyme-linked immunosorbent assay (ELISA)

Pre-inoculation (0 days, post-inoculation – days post-infection [dpi]) blood samples (0.3 ml each) were taken for control values. Blood samples were drawn through heart puncture from three rabbits per each experimental day. Serum levels of TNF-α and IFN-γ were measured by commercial enzyme-ELISA kits (MyBioSource, kit numbers MBS777411 and MBS777301, respectively). The levels of cytokines (pg/ml) were measured using a colorimetric reader (Stat Fax 303 Plus) and were calculated according to a cytokine standard curve supplied in the kits. Levels of cytokines IFN-γ and TNF-α were measured using a Sandwich ELISA method. All samples were tested in duplicate according to the manufacturer’s instructions.

### Detection of emperipolesis

Only engulfed nucleated cells or erythrocytes (not platelets) were considered as evidence of emperipolesis (Cashell and Buss [17]). These cells were then identified under the microscope and counted.

### Statistical analysis

Statistical tests were performed using SPSS (version 17.0 software package SPSS Inc., Chicago, IL, USA). Cell analysis and cytokine data were evaluated using the Student’s t-test. Differences between control and infection were considered significant when p-value was at least p<0.05.

## Results

### RHD pathology

Hypothermia, apathy, and dyspnea were all seen in the infected rabbits and they died 48-72 h post-infection. At autopsy, the rabbits showed typical features of RHD pathology. Liver necrosis and splenomegaly presented as primary manifestations of the disease. The liver was tan and mottled, with few reddish foci, and was brittle and easily torn. Hemorrhages were seen in the trachea, lungs, kidney capsule, and spleen. The lungs were highly edematous, the acute lung injury resulting from venous thrombosis leading to severe acute edema, frothy serous or bloody transudate that filled the trachea and permanently seeped from nostrils. While infarcts were characteristic for most organs, the kidneys were especially congested and colored dark brown.

### Changes in immune cell populations

Relative changes of cell populations in peripheral blood, spleen, BM, and lymph nodes are summarized in Tables-1-4.

**Table 1:** Blood cell counts in RHD.

Blood cells	Control	1 dpi	2 dpi	3 dpi
Lymphoblast	0.1±0.1	0.1±0.1	1.7±0.4*	2.1±0.5*
Lymphocyte	63.1±8.1	55.8±9.3	55.8±7.3	46.1±4.9**
Activated lymphocyte	0.3±0.1	7.6±2.6*	3.4±0.9*	7.9±2.1*
Myeloblast	-	0.1±0.1	0.3±0.1*	4.9±1.3*
Metamyelocyte	-	0.3±0.1	1.5±0.2*	3.6±1.0*
Band neutrophil	11.9±0.9	2.3±0.4**	4.0±0.5**	4.3±0.7**
Segmented neutrophil	19.3±2.2	20.3±3.8	18.9±4.2	14.3±3.5
Pathological neutrophil	-	-	2.8±0.8*	4.9±2.1*
Eosinophil	3.1±0.7	3.3±1.0	6.3±1.3*	4.5±1.2
Basophil	0.3±0.1	4.0±1.1*	2.6±0.9*	2.3±0.6*
Monoblast	-	-	1.2±0.3*	1.3±0.6*
Monocyte	1.9±0.5	5.4±1.3	1.5±0.2	3.8±0.9

* Significant increase compared with control (p<0.05, P<0.001).

** Significant decrease compared with control (p<0.05, P<0.001). RHD=Rabbit hemorrhagic disease, dpi=days post-infection

**Table 2:** Spleen cell populations in RHD.

Cell populations	Control	1 dpi	2 dpi	3 dpi
Erythroblast	0.9±0.1	7.2±0.8*	14.2±2.0*	4.2±1*
Lymphoblast	3.9±0.7	4.9±1.2	1.4±0.3**	3.2±0.7
Lymphocyte	83.8±4.2	71.4±6.3	64.6±5.8**	77.1±5.2
Lymphocyte activated	-	0.1±0.1	1±0.2*	2.1±0.7*
Myeloblast	0.3±0.1	0.3±0.1	4.5±0.4*	1.4±0.1*
Metamyelocyte	2±0.3	1±0.2	2.8±0.7	1.2±0.1
Band neutrophil	4.2±0.9	2.6±0.8	2±0.6**	1.9±0.3
Segmented neutrophil	1.5±0.4	1.9±0.4	1.4±0.5	2±0.4
Pathological neutrophil	-	0.1±0.1	0.2±0.1	0.1±0.1
Eosinophil	1.1±0.3	1.1±0.4	1.5±0.1	1.2±0.2
Basophil	0.1±0.1	0.2±0.1	0.4±0.2	2.2±0.4*
Monoblast	0.3±0.1	2.4±0.6*	3.2±0.7*	1.7±0.3*
Monocyte	1.7±0.2	6.1±0.9*	1.4±0.1	0.6±0.1
Macrophage	0.2±0.1	0.7±0.1*	1.4±0.2*	1.1±0.2*

* Significant increase compared with control (p<0.05, P<0.001).

** Significant decrease compared with control (p<0.05, P<0.001). RHD=Rabbit hemorrhagic disease, dpi=days post-infection

**Table 3:** Lymph node cell counts in RHD.

Cell population	Control	1 dpi	2 dpi	3 dpi
Erythroblast	-	-	0.8±0.1*	0.1±0.1
Lymphoblast	6.0±0.9	1.5±0.2**	6.1±0.8	2.9±0.5**
Lymphocyte	90.9±2.1	76.0±4.0**	76.5±5.2**	81.5±9.8
Lymphocyte activated	1.0±0.1	17.5±3.1**	2.2±1.0	0.9±0.2
Myeloblast	-	-	3.3±1.2*	4.6±0.8*
Metamyelocyte	-	1.0±0.2*	2.8±0.7*	3.0±0.8*
Band neutrophil	0.5±0.1	0.5±0.1	1.0±0.3	0.1±0.1
Segmented neutrophil	-	0.1±0.1	0.4±0.1*	0.1±0.1
Pathological neutrophil	-	-	-	0.4±0.1*
Eosinophil	0.1±0.1	0.1±0.1	0.1±0.1	0.1±0.1
Basophil	-	-	-	0.2±0.1
Monoblast	0.5±0.1	1.2±0.2	2.1±0.4*	1.3±0.3*
Monocyte	1.0±0.2	1.1±0.3	1.0±0.1	0.4±0.1
Macrophage	-	1.0±0.2	3.7±1.0*	4.4±0.7*

* Significant increase compared with control (p<0.05, P<0.001).

** Significant decrease compared with control (p<0.05, P<0.001). RHD=Rabbit hemorrhagic disease, dpi=days post-infection

**Table 4:** Bone marrow cell populations in RHD.

Cell populations	Control	1 dpi	2 dpi	3 dpi
Lymphoblast	5.7±0.8	0.7±0.3**	1.6±0.2**	2.1±0.3**
Lymphocyte	55.6±4.9	54.9±4.9	38.5±5.2**	40.3±2.8**
Lymphocyte activated	0.4±0.1	1.4±0.2	6.6±0.8*	5.9±1.1*
Large granular lymphocyte	0.2±0.1	0.2±0.1	1.0±0.3	1.1±0.1
Monoblast	2.0±0.3	1.8±0.6	1.6±0.4	1.9±0.5
Monocyte	2.2±0.8	2.0±0.7	2.1±0.3	1.3±0.5
Proerythroblast	0.4±0.1	3.2±0.8*	3.6±0.4*	1.6±0.2
Basophilic erythroblast	8.6±0.7	8.8±1.0	9.9±1.5	7.7±1.2
Late erythroblast	1.3±0.3	7.2±1.3*	16.8±0.9*	17.3±2.0*
Myeloblast	5.2±1.0	5.2±0.9	3.2±0.7**	1.9±0.4**
Metamyelocyte	8.9±1.8	4.4±1.2	3.1±0.8**	4.2±0.5**
Band neutrophil	5.7±1.0	4.1±1.2	3.1±0.4	4.3±0.5
Segmented neutrophil	2.2±0.5	2.5±0.4	-**	0.3±0.1**
Eosinophil	0.4±0.2	1.6±0.5	2.1±0.6	5.9±1.1
Macrophage	0.2±0.1	0.3±0.1	0.5±0.1	0.3±0.1
Megakaryocyte	0.4±0.1	0.7±0.2	0.5±0.2	1.1±0.3
Destructed cells	0.2±0.1	0.9±0.2	5.7±0.9*	2.9±0.6*

* Significant increase compared with control (p < 0.05, P < 0.001).

** Significant decrease compared with control (p < 0.05, P < 0.001). RHD = Rabbit hemorrhagic disease, dpi = days post-infection

#### Blood

**Figure-1:**
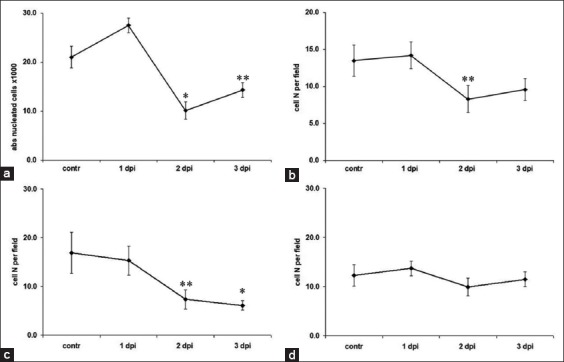
Absolute numbers of nucleated cells in the blood, lymph nodes, spleen, and bone marrow. (a) Blood, (b) lymph nodes, (c) spleen, (d) bone marrow. *A significant decrease compared with controls (p<0.05). **Tendency (p<0.1).

RHD is characterized by a rapid decrease of all the nucleated blood cells and subsequent pancytopenia observed by 2 dpi (Figure-1a). Early blast cell formation was seen as RHD progressed. The myeloid population that is normally represented by mature neutrophils (bands and segmented) was seen with metamyelocytes starting from 1^st^ dpi (Table-1). The count and percentages of mature neutrophils fell. The lymphoid cell populations showed many lymphoblasts (finely expressed nucleoli inside the nucleus) and activated lymphocytes with an altered nuclear-cytoplasmic ratio. Similar changes were observed in the monocyte population where monoblasts appeared after the 1^st^ dpi (Table-1), followed by a decrease in the total count of both monocytes and monoblasts.

Along with early developmental stages of cells, pathological cells also appeared in the blood of RHD animals. Pathological morphology was observed in neutrophils, primarily expressed in toxic granulations of the cytoplasm. Döhle bodies were seen in the cortical layer of neutrophil cytoplasm. Cellular vacuolization was also seen.

#### Spleen

Lymphoid cells were the main immune cells in the rabbit spleen consisting mainly of lymphocytes and few lymphoblasts (Table-2). Neutrophils comprised about 5% of the cells, with monocytes, and eosinophils and the basophil count was about 3%.

Progress of the disease was accompanied by a decrease of the lymphoid cell population leading to cytopenia (Table-2), with the lowest count being achieved by 2-3 dpi (Figure-1c). The decrease of lymphoid cell population paralleled the rise in myeloid cells by 2 dpi: Myeloblasts and metamyelocytes were morphologically the earliest detectable cells (Table-2). A decrease in the number of band neutrophils was accompanied by the development of a few pathological neutrophils as well as activated macrophages (foam cell).

#### Lymph nodes

The histology showed that the primary cells in the lymph node tissue were lymphocytes and lymphoblasts. Cytopenia was seen from 2 dpi. The terminal stage of the disease was characterized by the halving of lymphoblasts (Table-3). The decrease in total lymphocyte counts was accompanied by a marked increase in activated lymphocytes by 1 dpi followed by drastic fall and restoration by 3 dpi (Figure-1b).

A significant increase in myeloblasts and metamyelocytes was observed during the course of RHD (Table-3). Band neutrophils disappeared by 3 dpi, during the appearance of neutrophils showing pathological features. Monoblasts increased in number, with a parallel rise in macrophages.

#### BM

BM immune cells were mainly with lymphocytes, as well as lymphoblasts, metamyelocytes, and band neutrophils (Table-4). The erythroid population contained erythroblasts at different stages of maturity and a small population of proerythroblasts. The total number of BM cells did not change significantly during the course of RHD (Figure-1d); however, the immune population showed considerable alteration in its composition. Populations of lymphocytes, as well as lymphoblasts, metamyelocytes, and band neutrophils, tended to fall along with RHD progression (Table-4). The number of dead cells increased significantly (about 28 fold) during the first 2 days of infection and fell by a factor of 2 by the 3 dpi. It is worthy to note also the significant elevation of eosinophils and reduction of segmented neutrophils (Table-4).

### Pathological cells in peripheral blood

**Figure-2:**
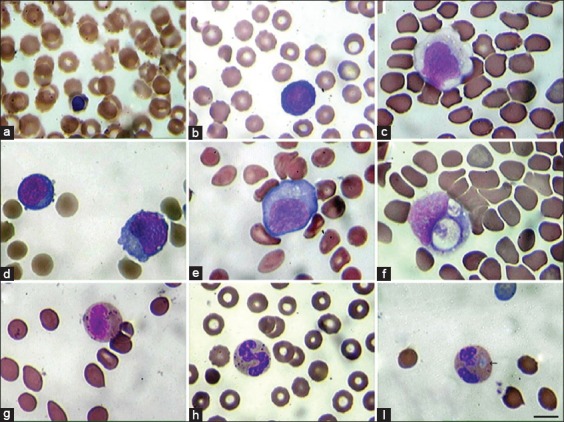
Pathological nucleatd cells in the peripheral blood in rabbit hemorrhagic disease. Cells were studied under light microscopy, 1250×, Giemsa Staining. (a) Orthochromatic erythroblast. (b) Basophilic erythroblast. (c) Monoblast with cytoplasmic vacuoles. (d) Normal and activated lymphocytes. (e) Lymphoblast. (f) Lymphocyte with cytoplasmic vacuoles and increased nuclear-cytoplasmic ratio. (g) Metamyelocyte. (h). Band neutrophil with pathological cytoplasmic granulations. (i) Neutrophil with Döhle body (arrow). Scale 10 µm.

Immature erythroid cells – erythroblasts – in the peripheral blood samples were seen from the start of the infection (Figure-2a and b). Cytoplasmic vacuoles were detected in monocytes and monoblasts (Figure-2c). Some activated lymphocytes were seen in the lymphoid cell population (Figure-2d). Furthermore, lymphoblasts (Figure-2e) and lymphocytes with cytoplasmic vacuoles and an increased nuclear-cytoplasmic ratio (Figure-2f) were observed. The neutrophil population expressed a left shift with a rise of metamyelocytes (Figure-2g) and neutrophils with pathological cytoplasmic granules (Figure-2h) and Döhle bodies (Figure-2i).

### RHDV cytoplasmic inclusions

**Figure-3:**
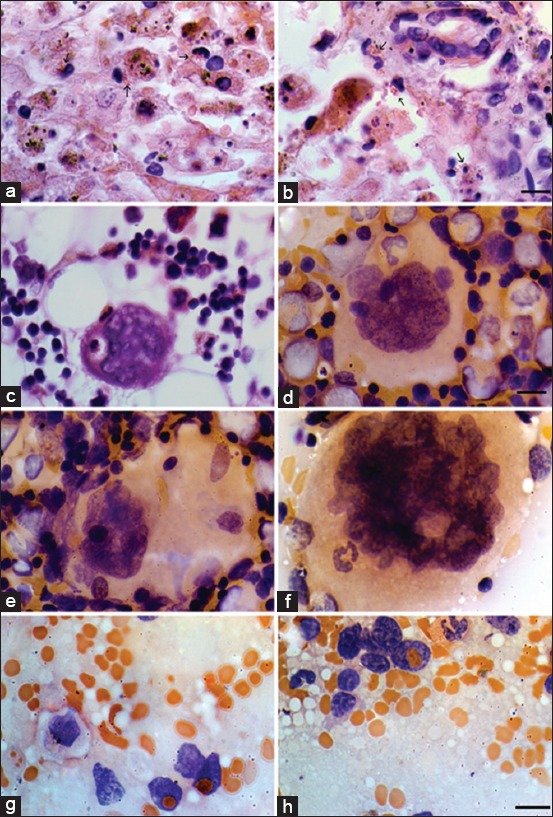
Rabbit hemorrhagic disease virus cytoplasmic inclusions and emperipolesis 72 h after infection (Hematoxylin and eosin staining). (a and b). Hepatocytes with massive necrosis and Kupffer cells with rabbit hemorrhagic disease virus (RHDV) cytoplasmic inclusions (arrows). Scale 20 µm. (c) Macrophage with RHDV eosinophilic cytoplasmic inclusions in a basophilic megakaryocyte (MKC), bone marrow. Scale 40 µm. (d) Lymphocyte is seen in an azurophilic MKC, bone marrow. Scale 40 µm. (e) Emperipolesis of lymphoid cells within azurophilic MKC, bone marrow. Scale 20 µm. (f) Band neutrophil inside an azurophilic MKC, bone marrow. Scale 20 µm. (g and h) Erythrocyte in the nucleus of lymphoid cells, spleen. Scale 20 µm.

On HE stained tissue sections, RHDV showed characteristic eosinophilic viral inclusions in the cytoplasm of infected cells (mainly macrophages) in highly variable amounts, rarely being totally absent. The main target cells were the hepatic phagocytes – Kupffer cells (Figure-3a and b), though the cytoplasmic inclusions were also observed in spleen and BM macrophages. RHDV inclusions varied in size generally from 0.2 to 0.5 µm. The number of cells containing cytoplasmic inclusions depended on virus infection dose (unpublished data).

### Emperipolesis

Ingested intact red blood cells and nucleated cells in megakaryocytes (MKCs) of the BM and spleen were common findings in RHD that provided evidence for megakaryocytic emperipolesis. It was observed in all samples of the BM MKC population, as well as spleen lymphoid cells (Figure-3c-h).

### Macrophage activation

At the terminal stage of RHD, an increase in the number of activated macrophages was observed in BM, spleen, and lymph nodes, but not in blood samples. Morphological alterations of these macrophages were seen as increased cytoplasmic volume (hypervacuolization) and phagocytized red blood cells, lymphocytes, and platelets (Figure-3g).

### Serum IFN-γ and TNF-α levels

**Figure-4:**
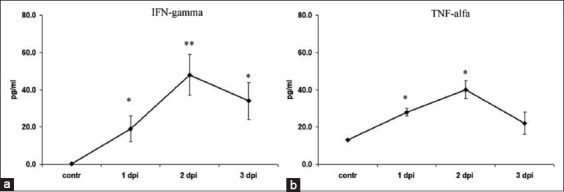
(a and b) Serum levels of interferon-alpha and tumor necrosis factor-gamma in rabbit hemorrhagic disease progression. *Significant increase compared with control (p<0.05). **Significant increase compared with control (p<0.01).

Serum IFN-γ was elevated in RHD infected animals, and its increase compared with controls was already significant by 1 dpi. The highest increase of IFN-γ was recorded by the end of 2 dpi (Figure-4a). Serum TNF-α reached maximal levels at 1-2 dpi (Figure-4b).

## Discussion

Some aspects of the pathological features of RHD are described by Vallejo *et al*. [7], Bonvehí *et al*. [18], Alexandrov *et al*. [19], Fuchs and Weissenbock [20], and Ferreira *et al*. [21]. However, little attention was paid to alterations in hematopoietic cell populations. This study of RHD immunopathology was focused on the cellular compositions of the blood, spleen, lymph nodes, and BM. It was shown that the absolute counts of cell populations were decreased with a marked left shift with the appearance and rise in early-stage and blast cells. Peripheral blood cell changes were accompanied by a decrease in all nucleated blood cells. The change in the myeloid cell population left shift was seen in the development of younger neutrophil populations – metamyelocytes, with more immature myeloid cells emerging. These cells were likely to have emerged from the BM, which showed a reduction in neutrophil precursors. This mostly resulted from increased tissue neutrophil demand and could be credited to either pathogen-induced neutrophil breakdown or neutrophil diapedesis [22].

BM cellular alterations were also expressed in an increased erythroblast population. In addition, nucleated erythroid cells developed and/or proliferated in spleen and lymph nodes. Such an increase was relative since it accompanied the fall in lymphoid cells. The increase in activated lymphocytes could be considered as a response to virus infection and viremia.

Cellular pathology was observed in neutrophils, lymphocytes, and macrophages. Neutrophil cytopathology was primarily expressed by toxic cytoplasmic granules. These degenerative changes in neutrophils may occur in some infectious diseases [23,24]. In addition, Döhle bodies were discovered in the cortical layer of the neutrophil cytoplasm, as well as in activated lymphocytes with increased nuclear-cytoplasmic ratio. Such pathological changes could be attributed to the reduction of maturation time in the process of new cell emergence [24]. Left shift, with inappropriate BM granulopoiesis, leads to immature neutrophil release [24]. The appearance of cytoplasmic toxicity may, therefore, serve as a marker of the disease, preceding the occurrence of quantitative leukogram abnormalities or the occurrence of left shift.

Pathological alterations also included macrophage hyperactivation, which was manifested as an increased macrophage count in various tissues, macrophage vacuolization (foam cell development), and phagocytosis of blood cells (red and white blood cells). A number of macrophages (mainly Kupffer cells, and rarely in spleen and BM) showed cytoplasmic eosinophilic inclusions characteristic of the reproductive cycle of many RNA viruses [25-27]. Finding of such inclusions followed a dose-dependent pattern (the higher the infection dose of the virus, the more frequent the inclusions). Although viral replication is presumed to occur predominantly in hepatocytes, and the GI.1 antigen can be detected in these cells as early as 12 h post-infection [7], viral antigens have also been detected in cells of the monocyte/macrophage lineage, including Kupffer cells and intravascular macrophages, most commonly in the lungs and spleen [9,19].

Infected hepatocytes initially show hydropic changes and ultimately become apoptotic and necrotic [20,21]. Necrotic foci coalesce as infection proceeds and this widespread hepatic damage leads to typical biochemical changes associated with hepatic disease, including marked elevations in serum transaminases and bilirubin [21].

RHD-induced hemorrhages are usually followed by an increase in thrombocyte counts, and MKCs congregate near and often invade across the endothelium to provide platelet delivery to blood flow. In terminal stages of differentiation, the MKCs develop compartmentalization of the cytoplasm into discrete thrombocytic zones [28]. The migratory pathway of neutrophils, in this case, is paved through demarcation system of MKCs, which may elucidate the emperipolesis described in the present research. This can be a result of the intensive release of hematopoietic cells from the BM in response to stress and increased body demand for these cells [29].

Our data indicate evidence of megakaryocytic emperipolesis in all infected rabbits from 24 h post-infection. Emperipolesis is a condition, where intact hematopoietic cells are seen in the cytoplasm of a host cell without any damage, both cells being viable [30,31]. The cells taken in frequently are neutrophils, lymphocytes, and plasma cells. The host cells may be MKCs, monocytes, endotheliocytes, fibroblast, and malignant cells [30,32]. BM uptake of neutrophils into MKCs can be interpreted as the morphological manifestation of megakaryocytic endocytosis [33] or as a conventional leukocyte migration – trans-megakaryocytic transport (transcytosis) across the vascular sinusoid wall tightly neighboring with an MKC from the adventitial lining [34,35].

Although the exact mechanism of emperipolesis is unknown, it has been hypothesized that the process requires free extracellular calcium, adhesive molecules, and an active cytoskeleton [36]. Alexandrov *et al*. demonstrated heterophilic leukocyte emperipolesis within the hepatocytes as the most prominent pathological feature found on electron microscopy in RHD pathology [37].

Quantitative cellular changes of rabbit lymphoid organs shown in our study were accompanied by elevation of specific inflammatory cytokines. The inflammatory process in RHD was characterized by the release of mediators involving inflammatory cytokines such as TNF-α and IFN-γ. Recent studies indicate a role of the immune response and specific cytokines, especially of peripheral blood leukocytes, in the pathogenesis of RHD [11,12]. Trzeciak-Ryczek *et al*. [11,12] demonstrated that the gene expression of the cytokines interleukin (IL)-6, IL-8, IL-10, TNF-α, TNF-β, IFN-γ, and granulocyte-macrophage colony-stimulating factor was increased in the peripheral blood leukocytes of RHDV-infected rabbits, and the level of expression depended on the course of RHD and affected the survival time of infected rabbits.

Hence, the main changes observed in immune cell populations as well as in cytopathology can be explained by an altered pro-inflammatory immune response.

## Conclusion

In RHD, there is a decreased absolute cell count in the blood as well as lymph nodes, spleen, and BM cell populations with a marked left shift toward more immature lineage representatives. It was seen as the rise of immature and blast cells. Quantitative cellular changes were accompanied by an elevation of specific inflammatory cytokines. Immunocytopathological alterations were observed as: Vacuolized, hyperactivated tissue macrophages, the finding of Döhle bodies in neutrophils, and development of activated lymphocytes with increased nuclear-cytoplasmic ratio. Cytoplasmic eosinophilic viral inclusions were found in tissue macrophages (liver, spleen, and BM) for the 1^st^ time in RHD. Emperipolesis was a common feature of RHD. Therefore, RHDV induces pathology in leukocytes expressed as hyperactivation with a shift macrophage count increased, the cells revealing viral inclusions with a cytopathic effect, as well as secretory activation (increased levels of pro-inflammatory cytokines).

## Authors’ Contributions

ABS, ZAK, and EMK designed the study. MAS, HHA, ZBS, NFK, and HSM performed the *in vivo* experiment and collected the samples. ASA, DMM, LHH, and LOA processed the immunological data. ZAK, ABS, and EMK analyzed the data. All authors read and approved the final manuscript.
